# Comparison of Commercial ELISA Kits to Confirm the Absence of Transmission in Malaria Elimination Settings

**DOI:** 10.3389/fpubh.2020.00480

**Published:** 2020-09-09

**Authors:** Lotus L. van den Hoogen, Paolo Bareng, Joana Alves, Ralph Reyes, Malou Macalinao, Júlio M. Rodrigues, José M. Fernandes, Lara F. Goméz, Tom Hall, Susheel K. Singh, Kimberly Fornace, Jennifer Luchavez, Alan Kitchen, Peter Chiodini, Fe Espino, Kevin K. A. Tetteh, Gillian Stresman, Nuno Sepúlveda, Chris Drakeley

**Affiliations:** ^1^Department of Infection Biology, London School of Hygiene and Tropical Medicine, London, United Kingdom; ^2^Department of Health, Research Institute for Tropical Medicine, Manila, Philippines; ^3^National Institute of Public Health, Praia, Cape Verde; ^4^Faculty of Science and Technology, University of Cape Verde, Praia, Cape Verde; ^5^Department of Natural, Life and Environmental Sciences, Jean Piaget University of Cape Verde, Praia, Cape Verde; ^6^Department of Congenital Disorders, Statens Serum Institut, Copenhagen, Denmark; ^7^Department of Immunology and Microbiology, Centre for Medical Parasitology, University of Copenhagen, Copenhagen, Denmark; ^8^NHS Blood and Transplant, London, United Kingdom; ^9^Hospital for Tropical Diseases and London School of Hygiene and Tropical Medicine, London, United Kingdom; ^10^Centre of Statistics and Applications, University of Lisbon, Lisbon, Portugal

**Keywords:** malaria, elimination, pre-elimination, ELISA, commercial ELISA kits, antibody, immunoglobulin, IgG

## Abstract

**Background:** Antimalarial antibody measurements are useful because they reflect historical and recent exposure to malaria. As such, they may provide additional information to assess ongoing transmission in low endemic or pre-elimination settings where cases are rare. In addition, the absence of antibody responses in certain individuals can indicate the cessation of transmission. Commercial malaria enzyme-linked immunosorbent assays (ELISA) detect antimalarial antibodies and are commonly used to screen blood donations for possible malaria infection. However, there is no standardized test to detect antimalarial antibodies for epidemiological use. Here we compared five commercially available ELISA kits (Trinity Biotech, newbio, DiaPro, Cellabs, and NovaTec) in search of a standardized tool for supporting claims of absence of malaria transmission. For comparison, a research-based (RB) ELISA protocol was performed alongside the commercial kits.

**Results:** The commercial kits were first compared using serum samples from known malaria-unexposed individuals (*n* = 223) and *Toxoplasma*-infected individuals (*n* = 191) to assess specificity and cross-reactivity against non-malaria infections. In addition, 134 samples from ≥10-year-olds collected in a hyperendemic region in the Gambia in the early 1990s were used to assess sensitivity. Three out of five kits showed high sensitivity (90–92%), high specificity (98–99%), low cross-reactivity (0–3%) and were considered user-friendly (Trinity Biotech, newbio and NovaTec). Two of these kits (Trinity Biotech and NovaTec) were taken forward for epidemiological evaluation and results were compared to those using the RB-ELISA. Samples from two pre-elimination settings (Praia, Cape Verde; *n* = 1,396, and Bataan, the Philippines; *n* = 1,824) were tested. Serological results from both the Trinity Biotech kit and the RB-ELISA concurred with recent passively detected case counts in both settings. Results from the Trinity Biotech kit reflected a significant decrease in the number of reported cases in Bataan in the 1990s better than the RB-ELISA. Results from the NovaTec kit did not reflect transmission patterns in either setting.

**Conclusion:** The Trinity Biotech commercial ELISA kit was considered reliable for epidemiological use and accurately described transmission patterns in two (previously) malaria endemic settings. The use of this simple and standardized serological tool may aid national control and elimination programs by confirming that regions are free from malaria.

## Introduction

A unique property of using antimalarial antibody responses as a measure of transmission is that when combined with age, they reflect the infection history of a given population (1–3). Antibody measures can therefore help to re-create transmission patterns over time. Evidence for any drop in, or absence of, antibodies can be interpreted as a decrease in malaria infections, or the complete cessation of transmission. The cumulative nature of exposure to malaria and its impact on the underlying antibody levels would result in smaller sample size needs to describe low rates of transmission compared to conventional metrics that use the proportion of infected individuals (4).

Historically, the absence of antibodies in children has been used as evidence of cessation of transmission in Greece and Mauritius (5, 6). In these studies, antibody responses to crude parasite extract were determined using an immunofluorescence antibody test (IFAT). More recently, studies from Aneityum and Iran suggested absence of transmission by assessing antibody responses to individual recombinant antigens (apical membrane antigen 1; AMA-1, and the 19 kDa fragment of merozoite surface protein 1; MSP-1_19_) (7, 8) or schizont extract (7) using an enzyme-linked immunosorbent assay (ELISA). Similar to the historical studies, children showed no antimalarial antibody responses, while some adults did have antimalarial antibodies owing to the persistence of antibodies (and/or memory B cells) once acquired (9–11). The ELISA platform is considered more objective than IFAT because antibody reactivity is determined by measuring optical density (OD) with a spectrophotometer rather than visual inspection of the strength of fluorescence using a fluorescence microscope (12). However, at present, there is no standardized ELISA protocol to measure malaria antibodies for epidemiological use. In particular, standard operating procedures, positive controls (i.e., hyperimmune sera) and negative controls (i.e., unexposed sera), as well as methods of normalization vary considerably between studies, which makes direct comparison of results between countries or populations challenging (4, 13).

There are several commercially available ELISA kits, for which production and operating procedures are standardized. Until now, these kits have been used to screen blood donations for evidence of malaria exposure prior to transfusion (14–20). In theory, these kits might be redeployed to an epidemiological context, as illustrated in studies from Ethiopia (21) and Iran (22). However, experimental evaluation of their use in describing malaria transmission in endemic settings is lacking. Therefore, we aimed to compare five of these commercially available ELISA kits for their applicability and performance in malaria epidemiology. We firstly assessed applicability by comparing assay characteristics such as ease-of-use, sensitivity, specificity, cross-reactivity and the amount of serum needed to test a sample. Secondly, we tested samples from two pre-elimination areas from Cape Verde (Praia) and the Philippines (Bataan). For comparison, a validated research-based ELISA protocol was performed alongside the commercial kits (1, 3, 23).

## Methods

### Study Population

#### Phase I: Technical Performance of the Commercial ELISA Kits

Assay performance was based on the proportion of samples correctly identified as negative using 223 samples from malaria unexposed UK donors (to assess specificity) as well as 191 samples from *Toxoplasma*-infected, malaria unexposed UK donors (to assess cross-reactivity). Malaria naivety was defined using a questionnaire to exclude malaria risk at the time of blood donation (14). *Toxoplasma* was diagnosed with nine commercially available *Toxoplasma* IgG and IgM tests (Supplementary Information) and was considered positive if it tested positive for any of these tests (J. Newham/A. Kitchen; *unpublished data*). To assess assay sensitivity, 134 samples collected from a hyperendemic region in the Gambia in the early 1990s were used. Sera were stored at −40°C until transportation on dry ice to the London School of Hygiene and Tropical Medicine (LSHTM) and stored at −80°C. Individuals were included only if they were 10 years or older by which exposure to malaria almost certainly would have occurred (24, 25). Furthermore, costs, the amount of serum needed to test a sample, and ease-of-use were assessed for all commercial kits and the research-based ELISA. For ease-of-use, a composite measure was created based on the number of incubation steps, total incubation time, need for sample preparation and whether reagents were ready-to-use.

#### Phase II: Epidemiological Performance of the Selected Commercial ELISA Kits

**Figure 1:**
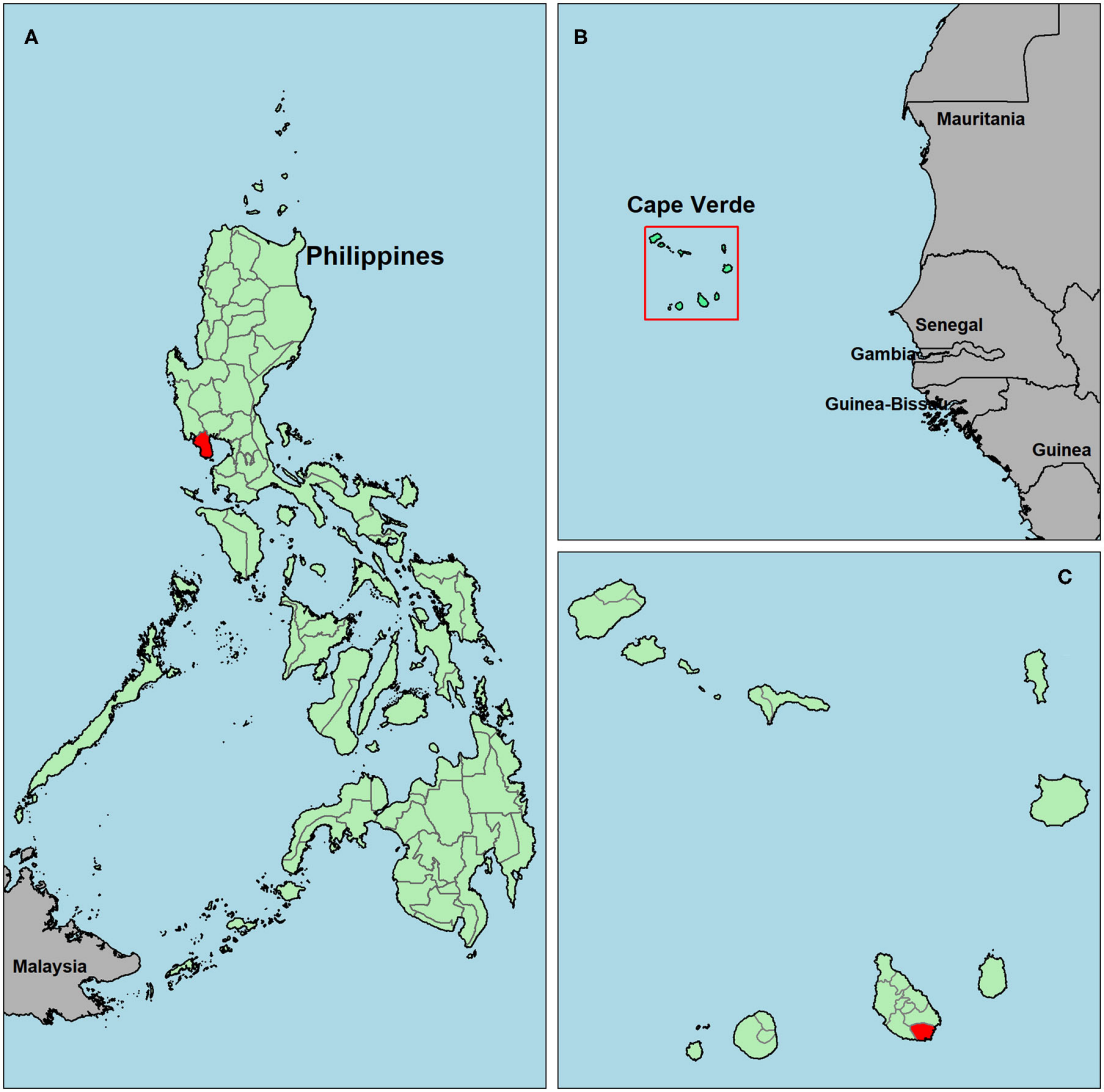
Map of survey locations in Bataan, the Philippines **(A)** and Praia, Cape Verde **(C)**. The square highlighting Cape Verde in **(B)** is enlarged in **(C)**. The Philippines **(A)** and Cape Verde **(B,C)** are shown in green, study areas are in red and surrounding countries are in gray.

Two study sites with contrasting malaria exposure histories and expected population immunity were selected. Firstly, samples were collected in Bataan, the Philippines in February 2017 (Figure 1A). In this setting, malaria incidence declined rapidly in the 1990s (26, 27) and it was declared malaria-free in October 2017 (28). Secondly, samples were collected in historical malaria hotspots of Praia, Cape Verde (Foton/Tira-Chapéu, Várzea/Taiti and Achada de Santo António; Figures 1B,C) which has seen unstable, low transmission since the late 1980s with occasional outbreaks (29–31). The most recent outbreak started mid-July 2017, with peak cases around the end of August and the end of October (32, 33). The majority of samples in the current study were collected before this outbreak (June-July 2017).

A two-stage cluster randomized sampling design was used with village or a sub-regional administrative unit as primary sampling unit and household as secondary sampling unit. A sample size of 2,000 individuals was initially specified for each setting in order to control the precision of the subsequent estimates of seroprevalence and seroconversion rates. Under a cross-sectional survey design, an entomological inoculation rate of 0.01 and the use of the MSP-1_19_ antigen, this sample size was expected to generate a 95% confidence interval for seroprevalence between 4.7% and 6.8% and for the seroconversion rate (SCR; the annual rate by which seronegative individuals become seropositive) between 0.0029 and 0.0043 for African settings, and 6.1 and 8.5% and 0.0029–0.0043 for non-African settings (34). This sample size also predicted a power >90% in detecting absence of malaria exposure for at least 3 years before data collection (35). For the study in Cape Verde, personnel and budget constraints led to a reduced sample size. The new sample size of ~1,500 individuals was predicted to decrease estimation precision slightly, with expected 95% confidence intervals between 4.5 and 7.0% for seroprevalence and ranging from 0.0028 to 0.0044 for the SCR.

Household members over 6 months old from randomly selected households were included in the analysis after consenting participation. A questionnaire was administered including demographic information and self-reported history of malaria. Up to 500 μl of whole blood from finger-prick were collected using microtainers with EDTA (Becton-Dickinson, Franklin Lakes, New Jersey). Serum was separated at collaborating institutions in-country and were stored at −20°C until shipment on dry ice to the London School of Hygiene and Tropical Medicine, London, UK. Serum was stored at −20°C until sample processing.

### Commercial ELISA Kits

Five commercial ELISA kits (Table 1) were used according to their standard protocols (Table 2): Trinity Biotech, newbio, DiaPro, Cellabs, and NovaTec. Customer services from both Trinity Biotech and Bio-Rad confirm that their malaria ELISA kit is the same. The Trinity Biotech or Bio-Rad kit is also known in the literature under previous distributers: Newmarket and Lab21. Optical density (OD) measures were read with a spectrophotometer (Dynex® Technologies) at a wavelength of 450 nm with a reference filter of 630 nm and OD measures were corrected for blank responses for DiaPro, Cellabs, and NovaTec kits (hereafter: OD_corr_) according to the instruction manuals.

**Table 1:** Assay characteristics for five commercial assays according to instruction manuals and the research-based enzyme-linked immunosorbent assay for antimalarial antibody detection.

	**Trinity biotech**	**newbio**	**DiaPro**	**Cellabs**	**NovaTec**	**Research-based**
Company	Trinity Biotech Plc, Wicklow, Ireland	Newmarket Biomedical Ltd, Suffolk, UK	Diagnostic Bioprobes Srl, Milan, Italy	Cellabs Pty Ltd., Brookvale, Australia	NovaTec Immundiagnostica GmbH, Dietzenbach, Germany	–
Antigenic targets	Four *Pf* and *Pv* recombinant antigens	Recombinant antigens	Recombinant proteins representing immunodominant epitopes	A panel of recombinant malaria antigens	*Pf* and *Pv* recombinant antigens	Five *Pf* recombinant proteins^a^
Detection of *Plasmodium* species^b^	*Pf, Pv, Pm, Po*	*Pf, Pv, Pm, Po*	*Plasmodium* species	*Pf, Pv, Pm, Po*	*Pf, Pv, Pm, Po*	*Pf*
Subclasses	IgG, IgM, IgA	Not reported	IgG, IgM, IgA	IgG	IgG, IgM	IgG
Samples/plate^c^	91	91	89	92	91	80
Duration	90 min	90 min	150 min	135 min	105 min	2.5 days
Plates/run	4	4	4	4	4	40
Specificity:						
Specificity from manual - all species *(n)*	96% (13,608)	100% (450)	>98% (NR)	100% (NR)	98% (NR)	–
Specificity from available literature^d^ (*n*, reference standard) (reference)	100% (8, malaria naive population) (20) 100%^g^ (50, malaria naive population) (15) 100%^e^ (17, malaria naive population) (36) 100%^g^ (23, malaria naive population) (37)		100% (8, malaria naive population) (20)	95% (58, IFAT) (38) 92% (50, malaria naive population) (15)	100% (8, malaria naive population) (20) 92% (245, microscopy) (39) 80% (96, microscopy) (40)	–
Sensitivity:						
Sensitivity from manual **-** all species *(n)*	NR	98% (528)	>95% (NR)	94% (NR)	96% (NR)	–
Sensitivity from manual - *Pf* only *(n)*	93% (76)	98% (410)	NR	NR	NR	–
Sensitivity from available literature (*n*, reference standard) (reference)	54%^e^ (56^f^, microscopy or history of malaria) (20) 55%^g^ (11, microscopy) (15) 53%^e^ (365, history of malaria) (36) 80%^d, g^ (45, microscopy and possible history of malaria) (37)		64% (56^e^, microscopy or history of malaria) (20)	71% (145, IFAT) (38) 91% (11, microscopy) (15)	55% (56^e^, microscopy or history of malaria) (20) 89% (245, microscopy) (39) 70% (88, microscopy) (40)	–

*Ig, immunoglobulin, min, minutes, Pf, Plasmodium falciparum; Pv, Plasmodium vivax; Pm, Plasmodium malariae; Po, Plasmodium ovale; n, samples tested; IFAT, immunofluorescence antibody test; NR, not reported*.

a *AMA-1, MSP1-19, MSP 2 Dd2, MSP2 CH150/9 and GLURP-R2 (for abbreviations and coating concentrations see methods section and Supplementary Information)*.

b *None of the instruction manuals of commercial kits mention detection of P. knowlesi*.

c *All assays use 96-well plates and require a certain number of controls to be run alongside samples*.

d *Some studies estimated specificity according to two methods: (1) using a different malaria diagnostic as the reference standard (e.g., microscopy or IFAT) or (2) using a malaria naive population (i.e., no travel history to a malaria endemic setting). In these cases, the specificity estimate using a naive population is presented here*.

e *Customer services from both Trinity Biotech and Bio-Rad confirm that their kits are the same*.

f *From 38 patients (i.e., includes multiple samples from the same participants); authors reported slight differences in the number of samples tested in each assay*.

g *Alternative names for the Trinity Biotech or Bio-Rad kit can be found in the literature: Newmarket and Lab21*.

**Table 2:** Standard operating procedures for five commercial kits and the research-based enzyme-linked immunosorbent assays for antimalarial antibody detection.

	**Trinity Biotech**	**newbio**	**DiaPro**	**Cellabs**	**NovaTec**	**Research-based**
Add sample (Dilution)	50 μl (neat)	50 μl (neat)	150 μl (3:4)	2 μl (1:100)	10 μl (1:101)	2 μl (1:1000)
Incubation, min (Temperature)	30 (37°C)	30 (37°C)	60 (37°C)	60 (RT)	60 (37°C)	Overnight (4°C)
Wash 1, *n*	5	5	4-5	4	3	5
Add conjugate	50 μL (prepare)	50 μL	#1: 150 μL (prepare) #2: 100 μL	100 μL (prepare)	100 μL	1:15,000 (prepare)
Incubation, min (Temperature)	30 (37°C)	30 (37°C)	#1: 30 #2: 30 (37°C)	60 (RT)	30 (RT)	180 (RT)
Wash 2, *n*	5	5	4–5	4	3	5
Add substrate	50 μL	50 μL	200 μL	100 μL (prepare)	100 μL	100 μL
Incubation (Temperature)	30 (RT)	30 (RT)	30 (RT)	15 (RT)	15 (RT)	15 (RT)
Add stop	50 μL	50 μL	100 μL	50 μL	100 μL	50 μL
Read plate	450 nm (reference 630 nm)	450 nm (reference 630 nm)	450 nm (reference 630 nm)	450 nm (blank on air)	450 nm (reference 630 nm)	450 nm

*RT, room temperature; min, minutes*.

### Research-Based ELISA

A previously described research-based (RB) ELISA protocol (1, 3, 23) was performed with the following modifications (Tables 1, 2). To maximize capture of antibody responses in a single assay, a pool of five *P. falciparum* antigens was used: apical membrane antigen 1 (AMA-1) (41), the 19 kDa fragment of merozoite surface protein 1 (MSP-1_19_) (42), the full-length Dd2 allele of MSP-2 (MSP-2 Dd2) (43), the full-length CH150/9 allele of MSP-2 (MSP-2 CH150/9) (44) and glutamate-rich protein R2 (GLURP-R2) (45). The coating concentration for all antigens was 0.5 μg/ml, except for GLURP-R2 which was coated at 0.1 μg/ml. To increase throughput and mimic commercial assays, samples were run in single wells. Samples were tested at a final concentration of 1:1,000. OD measures were read at a wavelength of 450 nm, corrected for blank responses (OD_corr_) and normalized using the standard curve as previously described (23). The standard methodology for the RB ELISA is included in the Supplementary Information.

### Statistical Analyses

All statistical analyses were performed in R version 3.4.1 (46). Antibody responses from infants under the age of 1 year old were removed due to possible presence of maternal antibodies. For the RB ELISA, a two-Gaussian mixture model was used to determine seropositivity (1). Seropositivity was defined at three standard deviations from the mean of the lowest Gaussian distribution of the mixture model. For commercial assays, thresholds for seropositivity were calculated according to manufacturer instruction manuals. For Phase II, results using seropositivity according to the mixture model approach were also explored for commercial kits. Histograms of OD values per assay and thresholds according to each method are provided in Supplementary Figure 1. Reversible catalytic models were fitted to the respective seroprevalence data adjusted for age. The models are parameterized by seroconversion and seroreversion rates (SCR and SRR, respectively), as described elsewhere (2, 3). Where visual examination suggested a change in transmission, a model assuming two age-related SCRs was run and fitting tested by likelihood ratio tests (1, 7).

The following serological metrics were used to show the presence of low-level recent transmission in Cape Verde and the absence of recent transmission in the Philippines: seroprevalence in children aged 1–5 years, SCRs and, where applicable, the presence of a change point in transmission. In addition, 95% Pearson-Clopper (exact) confidence intervals (CI) were used to quantify the uncertainty associated with seroprevalence. Pearson's χ^2^ tests for two-way contingency tables were used to compare seroprevalence among individuals who reported a history of malaria and those who did not; Fisher Exact Tests were used when the counts were below 5. Previous reports on malaria outbreaks in Cape Verde have identified malaria risk to be highest in adult men: more than two-thirds of the passively detected cases between 2007 and 2016 were in men older than 20 years (possibly due to occupational or travel-related risk) (29, 30). Therefore, seroprevalence in children aged 1 to 5 years was not used as a metric for absence/presence of malaria in this setting. Instead, malaria risk in adult men was assessed by comparing seroconversion models with one force of infection to those with a change point at age 20 for men (*n* = 563) and women (*n* = 860) separately. A 5% significance level was used throughout the paper.

## Results

### Phase I: Technical Performance of the Commercial ELISA Kits

Sensitivity, testing sera from individuals in a hyperendemic region in the Gambia in the 1990s as true positives, was high (90–95%) except for the DiaPro kit (86%); Table 3. Specificity using samples from either malaria unexposed individuals or those infected by *Toxoplasma* was high across most kits (>96%) except for the Cellabs kit (81 and 84%, respectively). Receiver operating characteristic (ROC) curves for sensitivity and specificity estimates are included in Supplementary Figure 2. Costs per sample were highest for the DiaPro, Cellabs, and NovaTec kits (>$2 USD compared to < $2 USD for the Trinity Biotech and newbio kits), while the DiaPro kit needed the highest volume of serum (150 μl compared to ≤ 50 μl for the other kits) and was considered least user-friendly (Table 3). Additionally, commercial production of the newbio kit was discontinued by the manufacturer after the finalization of Phase I. Based on these considerations, the Trinity Biotech and NovaTec kits were taken forward for evaluation with epidemiological samples.

**Table 3:** Cost per sample, amount of serum needed to run a sample, ease-of-use, specificity, and cross-reactivity for five commercial kits and the research-based enzyme-linked immunosorbent assay for antimalarial antibody detection.

	**Trinity Biotech**	**newbio**	**DiaPro**	**Cellabs**	**NovaTec**	**Research-based^b^**
Approximate cost/sample^a^	$1.59	$1.92	$2.68	$3.04	$2.09	$0.25
Amount of sample	50 μL	50 μL	150 μL	2 μL	10 μL	2 μL
Ease-of-useSample preparation Incubation steps Incubation time Ready-to-use reagents (n/N)	High No 3 90 min 2/3	High No 3 90 min 3/3	Low Yes 4 150 min 3/4	Medium Yes 3 135 min 1/3	High Yes 3 105 min 3/3	– Yes 3 2x overnight 2/3
Proportion negative for:						
Malaria-naive (*n* = 223)	99% (97–100%)	99% (97–100%)	98% (95–99%)	81% (75–86%)	98% (95–100%)	–
*Toxoplasma*-exposed and malaria-naive (*n* = 191)	100% (98–100%)	99% (96–100%)	100% (98–100%)	84% (78–89%)	98% (95–99%)	–
Proportion positive for:						
Malaria-exposed^c^ (*n* = 134)	92% (88–95%)	91% (86–94%)	86% (81–91)	95% (91–97%)	90% (85–93%)	–

a *Costs per sample are based on running a 96-well plate of samples except for wells allocated for necessary controls (see Table 1); it does not include technician time. Commercial assays were bought in bulk (i.e. 25 plates per brand) in January 2016 for Phase I and March 2017 for Phase II. Costs shown are based on the most recent prices for Trinity Biotech and NovaTec*.

b *Evaluation of assay performance (Phase I) focused on the commercial assays, however, where available, information for the research-based assay is included for reference*.

c *Sera were collected in a hyperendemic region in the Gambia in the early 1990s and individuals were only included if they were 10 years or older by when exposure to malaria almost certainly would have occurred (24, 25)*.

### Phase II: Epidemiological Evaluation of the Selected Commercial ELISA Kits

#### Bataan, the Philippines

**Figure 2:**
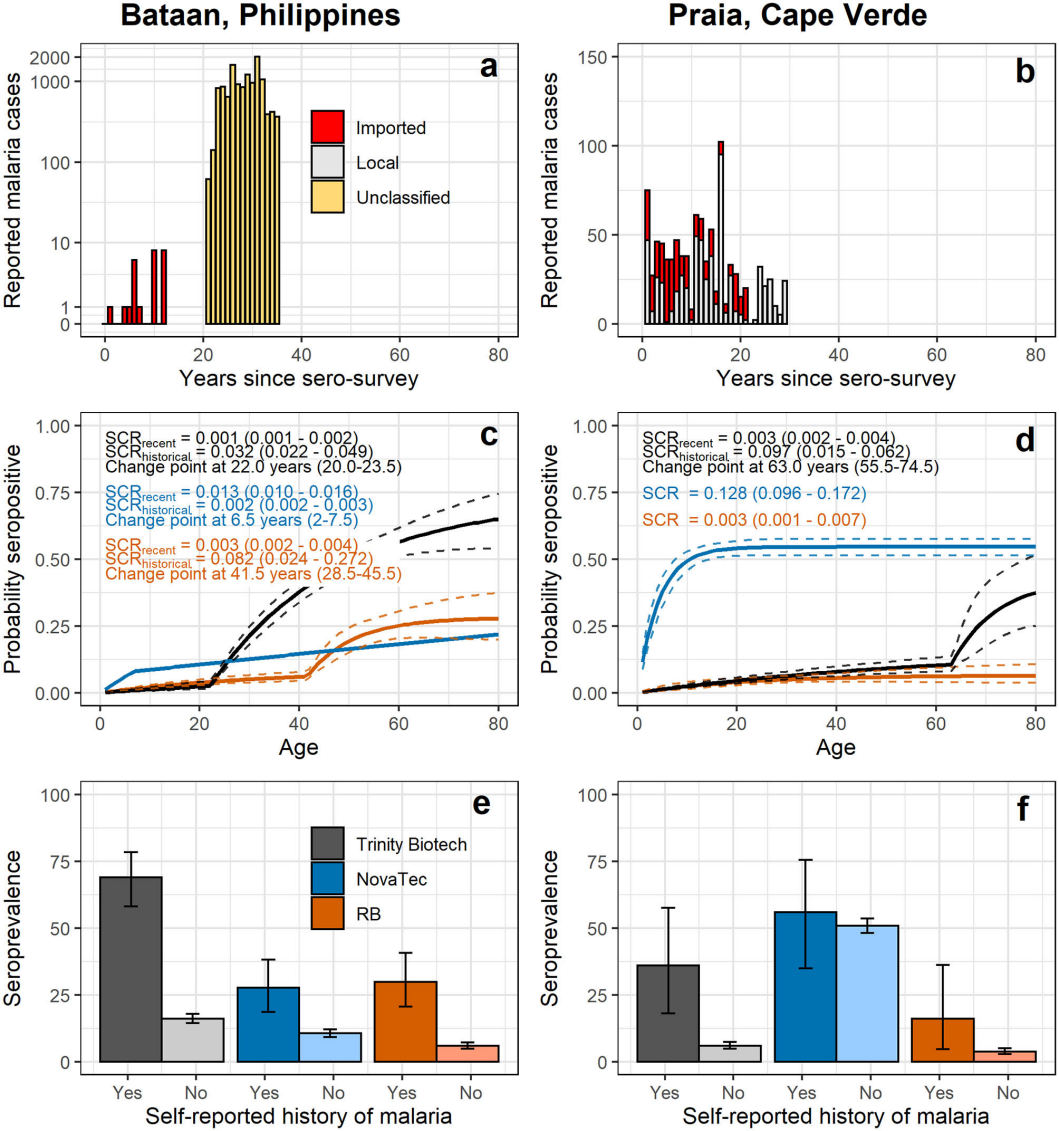
Reported malaria cases **(a,b)**, seroconversion curves **(c,d)** and seroprevalence by self-reported history of malaria **(e,f)** using antibody responses recorded by commercial and the research-based (RB) enzyme-linked immunosorbent assays. In **(a,b)** counts of reported malaria cases at local health facilities are shown over time; in Bataan, data was available for 0 to 12 years prior to data collection (i.e., 2017 – 2005) (28) and 21–35 years prior to data collection (i.e., 1996 – 1982) (26). In Praia, data was available from 1 to 21 years prior to data collection (i.e., 2016 – 1996) (30) and 22 to 31 years prior to data collection (i.e., 1995 – 1986) (29). In **(c,d)** seroconversion curves of age-specific seroprevalence are shown; solid lines represent the fit of the reversible catalytic model (2), while dashed lines represent 95% confidence intervals (CIs). Seroconversion rate and change point estimates with 95% CIs are shown on plots. In **(e,f)** seroprevalence estimates and 95% CIs are shown by self-reported history of malaria. Results for commercial kits using a two-Gaussian mixture model for seropositivity thresholds are shown in Supplementary Figure 4.

**Figure 3:**
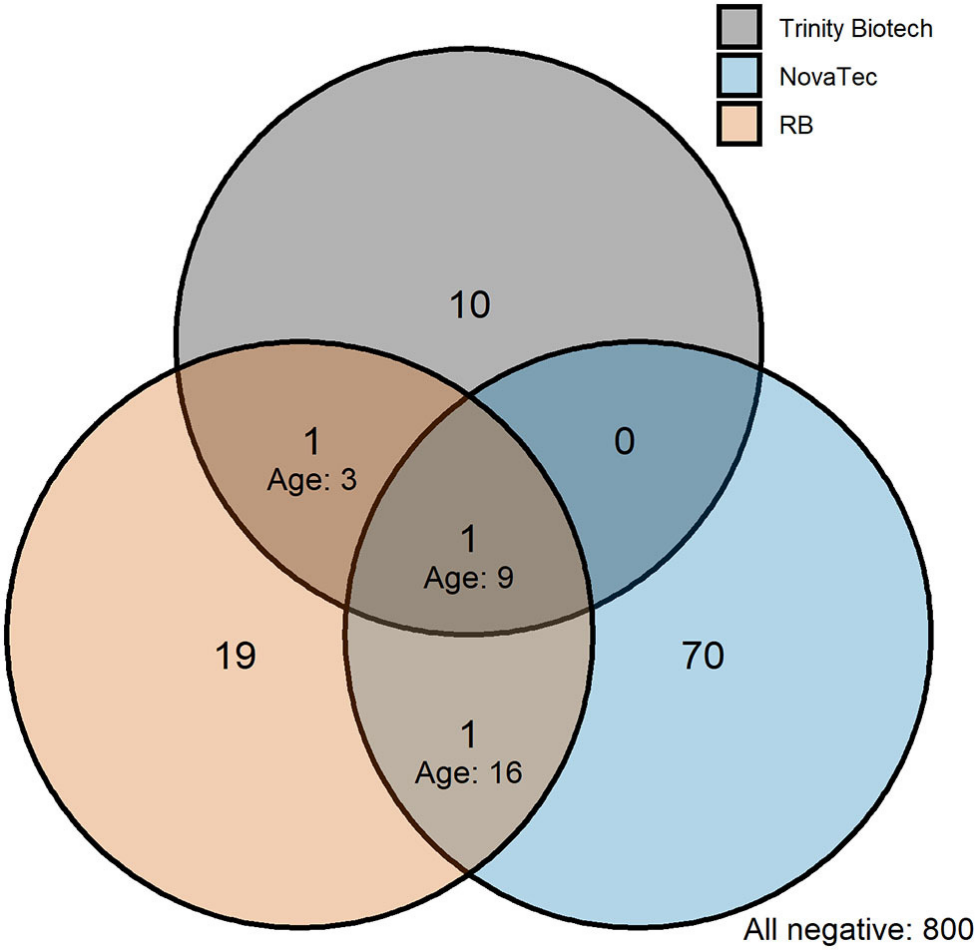
Venn diagram showing the intersection of seropositivity recorded by commercial and the research-based (RB) enzyme-linked immunosorbent assays in those born since the decline in passively detected malaria cases in Bataan, the Philippines. Seropositivity is shown for those younger than 22 years corresponding to the marked decline in passively detected cases in 1995, see Figure 2a. Ages are shown on the plot for individuals who were seropositive in multiple assays.

In Bataan, the Philippines, 1,824 out of 2,050 samples collected were available (Table 4). Seroprevalence in 236 children 1 to 5 years old was 1.7% (*n* = 4; 95% CI: 0.4–4.3%) for the Trinity Biotech kit; 4.7% (*n* = 11; 2.3–8.2%) for the NovaTec kit; and 1.7% (*n* = 4; 0.4–4.3%) for the RB-ELISA. The best fit seroconversion curve for the Trinity Biotech kit showed a change point at 22.0 years (95% CI: 20.0–23.5, *p* < 0.001; Figure 2b), which coincides with the marked decrease in locally reported cases ~22 years previously (Figure 2a). The RB-ELISA also showed a change in the seroconversion curve (*p* < 0.001), though estimated at 41.5 years ago with a wider 95% confidence interval (28.5–45.5). The SCR estimates ranged from 0.001 annual seroconversion events per person (95% CI: 0.001–0.002) for the Trinity Biotech kit; 0.003 (0.002–0.004) for the RB-ELISA; and 0.013 (0.010–0.016) for the NovaTec kit. For all assays, seroprevalence in those with self-reported history of malaria (*n* = 87) was higher compared to those without (*p* < 0.001) but this difference was greatest for the Trinity Biotech kit (Figure 2c). Continuous OD measurements before and after the detected change point showed marked differentiation for the Trinity Biotech kit but less so for the NovaTec kit and the RB-ELISA (Supplementary Figure 3). Seropositivity in those younger than 22 years (i.e., those born since the marked decrease in reported cases in 1995) was low for the Trinity Biotech kit (1.3%, 12/902; 0.7–2.3%) and the RB-ELISA (2.4%, 22/902; 1.5–3.7%), compared to the NovaTec kit (8.0%, 72/902; 6.3–10.0%). Antibody levels in these seropositives were relatively weak (i.e., OD < 1) for the Trinity Biotech kit, except for one child (OD: 2.523; age: 12). For the RB-ELISA, only three had an OD_corr_ > 1 (ages: 9, 6 and 3) and for the NovaTec kit this was twelve (age range: 1–18). Very few were seropositive in multiple assays (*n* = 3, ages 3, 9 and 16; Figure 3) and none had an OD/OD_corr_ > 1 in multiple assays.

**Table 4:** General characteristics of the study population in Bataan, the Philippines and Praia, Cape Verde.

	**Bataan, the Philippines**	**Praia, Cape Verde**
Number of samples collected	2,050	1,432
- Number of samples analyzed in assays - No age data available or <1-year-old - Not enough serum available for all tests Lived outside of main study area	1,824 11 179 36	1,396 33 11 N/A
Age group, % (*n*)		
−1 to 5 - 6 to 15 - >15	12.9% (236) 26.0% (475) 61.0% (1,113)	8.3% (116) 22.9% (319) 68.8% (961)
Self-reported history of malaria, % (n/N) *Youngest participant with self-reported history of malaria*	4.8% (87/1,812) *10 years old*	1.8% (25/1,394) *16 years old*

#### Praia, Cape Verde

In Cape Verde, 1,396 out of 1,432 samples collected were available for analyses (Table 4). For the Trinity Biotech kit and the RB-ELISA, (recent) SCRs were low (Figure 2e), consistent with historically low case counts (Figure 2d), with 0.003 annual seroconversion events per person for each assay. The SCR for the NovaTec kit was high (0.128 annual seroconversion events per person, 0.096–0.172), but when using the mixture model approach to define seropositivity instead of the kit-based threshold, the SCR was lower (0.032, 0.022–0.048; Supplementary Figure 4). Few participants reported a history of malaria (*n* = 25; Table 4). Seroprevalence was higher in those who did vs. those who did not for both the Trinity Biotech kit (odds ratio, OR, 8.91, 95% CI: 3.68–20.47; *p* < 0.001) and the RB-ELISA (OR 4.82, 1.37–13.22; *p* = 0.005). This difference was not statistically significant for the NovaTec kit (OR 1.23, 0.56–2.79; *p* = 0.612; Figure 2f). Both the RB-ELISA and the Trinity Biotech kit showed evidence of an increased SCR in adult men (i.e., *p* = 0.060 and *p* = 0.026 comparing models with and without a change point at 20 years for men only; Supplementary Table 1). For women as well as men and women combined, this was not seen. Using mixture models to define the seropositivity threshold, there was some evidence for a higher SCR in those aged <2–3 years using the Trinity Biotech kit (0.020 vs. 0.003; *p* = 0.009) and the RB-ELISA (0.007 vs. 0.001; *p* = 0.090), Supplementary Table 1. However, in both cases, this was based on only 1 seropositive infant and ODs were low (Trinity Biotech kit: 1/26 infants aged 1-2, OD 0.071; RB-ELISA: 1/54 infants aged 1-3, OD_corr_ 0.372). For the NovaTec kit, the SCR was zero for those aged 3 or less but with a wide 95% CI (2.0–15.0 years; Supplementary Table 1).

## Discussion

There is historical evidence that demonstrating the absence of specific antibody responses can contribute to verifying areas or populations as malaria-free (5, 6), but there is no standardized approach available. Commercially available ELISA kits undergo rigid standardization processes and have been applied to screen blood products prior to donation to minimize risks of transfusion-transmitted malaria (14–20). Here, we compared five commercial kits alongside a research-based (RB) ELISA for their technical (Phase I) and epidemiological (Phase II) performance in characterization of malaria transmission at low endemicity and pre-elimination. In technical performance assessments, three kits were discounted from further analysis due to poor sensitivity (DiaPro), poor specificity (Cellabs), high cross-reactivity to *Toxoplasma gondii* (Cellabs), low ease-of-use (DiaPro), high required blood volume (DiaPro) or production being discontinued (newbio). Further evaluation of kits in epidemiological characterization with samples from the Philippines and Cape Verde found that the Trinity Biotech kit described historical and recent malaria transmission patterns most accurately.

Firstly, we used serum samples from a hyperendemic region and those from malaria naive populations to assess sensitivity and specificity. Specificity estimates for the kits reported here and from previous studies were largely similar to those reported in instruction manuals (Tables 1, 3). Specificity was slightly lower where previous studies used samples from endemic settings and microscopy was the reference standard, as opposed to using samples from a malaria naive population. Sensitivity estimates from the available literature were considerably lower than those presented by us and instruction manuals. This may be due to the fact that previous studies have used microscopy or IFAT positivity as the reference standard and/or samples from non-endemic settings (i.e., returning travelers with a clinical history of malaria), whereas we used sera collected in a hyperendemic region. Overall, this highlights the need for a reliable reference standard (i.e., confirmed recent infection) to generate estimates for these and other serological assays.

Our rationale for extending technical performance evaluation with ease-of-use and costs was that whilst ELISA kits used for blood screening may have undergone some level of standardization, their intended use case scenario is different from epidemiological screening. Kits are typically designed to test batches of dozens to hundreds of samples, whereas a study to verify the absence of malaria transmission may include hundreds (7) or even thousands of participants (47). Ease-of-use, cost and scalability to large epidemiological studies are thus important considerations. Whilst rudimentary, our screen found that some assays were significantly more protracted to complete than others and used higher blood volumes than those that might be collected in epidemiological surveys. Blood collection in surveys is typically done via fingerprick resulting in 50–500 μl sample volumes depending on age and/or compliance and may be stored as separated liquid plasma/serum or dried onto filter papers (Dried Blood Spot, DBS). Excision and elution of DBSs is time consuming but can outweigh the need for cold chain which has significant practical and logistical advantages in field surveys (23). It was for this latter reason that we included a research-based (RB) ELISA which has been developed, and extensively tested, for use with DBS samples (1, 3, 23).

Although this was not an exhaustive assessment of all currently available commercial ELISA kits for antimalarial antibody detection, one of the five kits showed significant promise in correlating with described transmission patterns in two endemic settings. In Bataan, The Philippines, seroconversion curve profiles generated from the Trinity Biotech kit corresponded with a decrease in passively detected malaria cases in 1995 (26). Seroprevalence in those born since this decrease in transmission was low (1.3%). Whether these represent false-positive results or true responses following asymptomatic, low-density infections or infections acquired outside the study area, is unknown. However, ODs were relatively low and very few were positive in multiple assays which suggests that these are false-positive observations. Additionally, the current estimate of seroconversion was low in Bataan, 0.001 annual seroconversion events per person, which is lower than that recorded in Sri Lanka during pre-elimination using a RB-ELISA protocol (48). Bataan was declared malaria-free shortly after samples were collected for this study (28). Although the RB-ELISA detected a change point in transmission in the site in the Philippines, this preceded the drop in reported cases to local health facilities (including the lower estimate from the 95% confidence interval for the change point). This is perhaps a result of decreased sensitivity due to the higher sample dilution (1:1000 for the RB-ELISA vs. neat for the Trinity Biotech kit) or a (previous) behavior-related risk of exposure to malaria. Lastly, the decreased sensitivity could have been caused by the RB-ELISA detecting *P. falciparum* alone, while the commercial kits additionally detect non-*falciparum* species (except for *P. knowlesi*). Historically, *P. vivax* was also present in Bataan like much of the Philippines.

For Cape Verde, the low levels of transmission over the past decades were correctly identified as shown by the low, constant SCR recorded by the Trinity Biotech kit (i.e., 0.003 annual seroconversion events per person). The Trinity Biotech kit also identified a higher exposure to malaria in adult men (i.e., >20 years old compared to those younger than 20 years old) as previously described in epidemiological studies in Cape Verde (29, 30). Seroconversion curves generated from results from the RB-ELISA were similar to those for the Trinity Biotech kit in Cape Verde. Results from the NovaTec kit did not reflect malaria transmission patterns in either setting and therefore it probably has limited utility for epidemiological characterization of transmission.

The dynamic range of OD values recorded in endemic populations was greatest for the Trinity Biotech kit and despite the use of neat serum, little to no background responses were seen (Supplementary Figure 3), thus blocking of non-specific binding to malaria antigens seems extremely efficient. Another advantage of commercial kits over the RB-ELISA is that both kits (Trinity Biotech and NovaTec) report detection of IgM and IgG, while the RB-ELISA detects only total IgG, and IgM may be more informative in representing a recent infection. An optimal assay would be refined to use DBS whilst maintaining high accuracy together with reducing costs (i.e., currently >$1.50/sample for commercial kits vs. approximately $0.25/sample for the RB-ELISA, excluding technician time). An overview of these and other outstanding technological refinements for assays as well as programmatic questions that need to be addressed are shown in Box 1.

Box 1 **Future work: Technological refinements of the antimalarial antibody detection assays and programmatic questions that need to be addressed for their use in confirmation of malaria elimination.**

Technological refinements

- Broaden assessments of sensitivity and specificity from non-malaria infections

- Optimize assays for use with dried blood spots

- Evaluate options for scenario-specific assays by selecting antibodies with known exposure profiles (i.e., recent vs. any) or specific Ig class responses

- Confirm suitability for non-*Plasmodium falciparum* species

- Evaluate best methodology to determine seropositivity at (very) low transmission

Programmatic questions

- Revisit costs per sample (aiming for <1 USD/sample)

- Establish limits of non-specific reaction (i.e., false-positivity rate)

- Establish sampling frames for specific epidemiological scenarios and disease transmission patterns; i.e., determine who and where to sample to confirm absence of malaria transmission (with consideration of age- and/or behavior-related risk of malaria)

- Improve understanding of relationship between seroprevalence, SCR and annual parasite incidence (API)/entomological inoculation rate (EIR) at (very) low transmission

## Conclusion

The Trinity Biotech commercial ELISA kit was considered most applicable for large-scale use in epidemiological surveys and accurately described malaria transmission in two pre-elimination settings. All the commercial ELISA kits studied reported the detection of four human malaria species: *P*. *falciparum, P*. *vivax, P*. *malariae*, and *P*. *ovale*, but the accuracy of these assays in detecting exposure to *P. knowlesi* is unknown. The performance of the kits using DBS samples remains to be evaluated. Future work should focus on these technological refinements as well as outstanding programmatic questions relevant to the use of serological tools for certification of malaria-free populations.

## Data Availability Statement

The datasets generated for this study are available on request to the corresponding author.

## Ethics Statement

The studies involving human participants were reviewed and approved by the LSHTM Research Ethics Committee (11684) for testing of anonymised UK donor samples collected by Public Health England/NHS Blood and Transplant. For the surveys in Cape Verde and the Philippines, ethical approval was obtained through the LSHTM Research Ethics Committee (11684), the Ethics Committee for Research in Health in Cape Verde (65/2016) and the Research Institute for Tropical Medicine in the Philippines (2016-26). Written informed consent to participate in this study was provided by the participants' legal guardian/next of kin.

## Author Contributions

CD, GS, NS, KT, AK, PC, and LH conceived the study. CD, GS, NS, KF, and FE designed the surveys. PB, JA, RR, MM, KF, JL, JF, JR, LG, NS, and GS performed field data collection. KT and SS provided antigen constructs. LH performed laboratory data collection with support from KT and TH. LH and CD analyzed and interpreted the data. LH wrote the first draft of the manuscript with support from CD. All authors read and approved the final manuscript.

## Conflict of Interest

The authors declare that the research was conducted in the absence of any commercial or financial relationships that could be construed as a potential conflict of interest. The reviewer DN declared a shared affiliation, though no other collaboration, with KT and CD to the handling Editor.
