# Supplementary material for: Comparison of Commercial ELISA Kits to Confirm the Absence of Transmission in Malaria Elimination Settings
Source: Front Public Health. 2020 Sep 9;8:480. doi: 10.3389/fpubh.2020.00480 (PMC7509087; doi:10.3389/fpubh.2020.00480)
Supplement: Supplementary file 1 [file Data_Sheet_1.docx]

**Supplementary Data**

*Toxoplasma* diagnosis……………………………………………………………………. Page 2

Standard methodology research-based enzyme-linked immunosorbent assay…………... Page 3

Supplementary Figures & Tables………………………………………………………… Page 4-8

***Toxoplasma* diagnosis**

*Toxoplasma* was diagnosed with nine commercially available *Toxoplasma* IgG and IgM tests. All 191 serum samples were tested on:

- Microgen Mercia Toxoplasma IgG
- Microgen Mercia Toxoplasma IgM
- Biorad Platelia Toxoplasma IgG
- Biorad Platelia Toxoplasma IgM
- Access Toxoplasma IgG
- Access Toxoplasma IgM
- Mast Diagnostics Mastafluor Toxoplasma IgG
- Mast Diagnostics Mastafluor Toxoplasma IgM
- Euroimmune Toxoplasma IgG ELISA
- Euroimmune Toxoplasma IgG IFT
- Euroimmune Toxoplasma IgM ELISA
- Euroimmune Toxoplasma IgM IFT
- Biokit Toxocell Latex
- Abbott Architect Toxoplasma IgG
- Abbott Architect Toxoplasma IgG Avidity
- Abbott Architect Toxoplasma IgM
- Diasorin Liason Toxoplasma IgG II
- Biomerieux Vidas Toxoplasma IgG II
- Biomerieux Vidas Toxoplasma IgG Avidity
- Biomerieux Vidas Toxoplasma IgM
- Biomerieux Vidas Toxoplasma Competition

A sample was considered positive if it tested positive for any of these tests. A subset of samples was confirmed by the Sabin Feldman dye test and the IgM immunosorbent agglutination assay at the Swansea Toxoplasma Reference Laboratory.

J. Newham/A. Kitchen; *unpublished data*

**Standard methodology research-based enzyme-linked immunosorbent assay**

Participants’ sera were diluted to a concentration of 1:200 in phosphate buffered saline with 0.5% Tween20 (PBS-T) and 0.05% sodium azide. A pool of five *Plasmodium falciparum* antigens was used: apical membrane antigen 1 (AMA-1), the 19 kDa fragment of merozoite surface protein 1 (MSP-1_19_), the full-length Dd2 allele of MSP-2 (MSP-2 Dd2), the full-length CH150/9 allele of MSP-2 (MSP-2 CH150/9) and glutamate-rich protein R2 (GLURP-R2). Antigens were coated overnight to plates (Immulon 4HBX flat bottom, Thermo) using coating buffer (50 µl per well; 0.2 M NaHCO_3_/Na_2_CO_3_, pH 9.4-9.6). The coating concentration for all antigens was 0.5 µg/ml, except for GLURP-R2 which was coated at 0.1 µg/ml. The next day, plates were washed 3 times with wash buffer (PBS-T) and incubated with blocking buffer for three hours (150 µl per well; 1% skimmed milk powder in PBS-T). Next, after 3 washes, samples were diluted into plates at 1:5 in blocking buffer, thus generating a final serum concentration of 1:1,000. To increase throughput and mimic commercial assays, samples were run in single wells. A positive control standard curve using a Tanzanian hyperimmune sera pool (not commercially available) was created fresh for each batch of plates. The standard curve was created by a 6-fold serial dilution series over 6 wells starting at 1:50. Standard curves were run in duplicate on each plate. Lastly, four wells were left blank, i.e. these were incubated with blocking buffer only. On the third day, after 5 washes, plates were incubated with secondary antibody at 1:15,000 in blocking buffer for three hours (50 µl per well; horseradish peroxidase-conjugated rabbit anti-human IgG, #P0214, Dako). After a final 3 washes, plates were developed using TMB (100 µl per well; #TMBW-1000-01, Tebu-bio laboratories) for 15 minutes in the dark and then stopped using 0.2 M H_2_SO_4_ (50 µl per well). OD measures were read at a wavelength of 450nm with a spectrophotometer (Dynex® Technologies).

**Supplementary Figures & Tables**

**
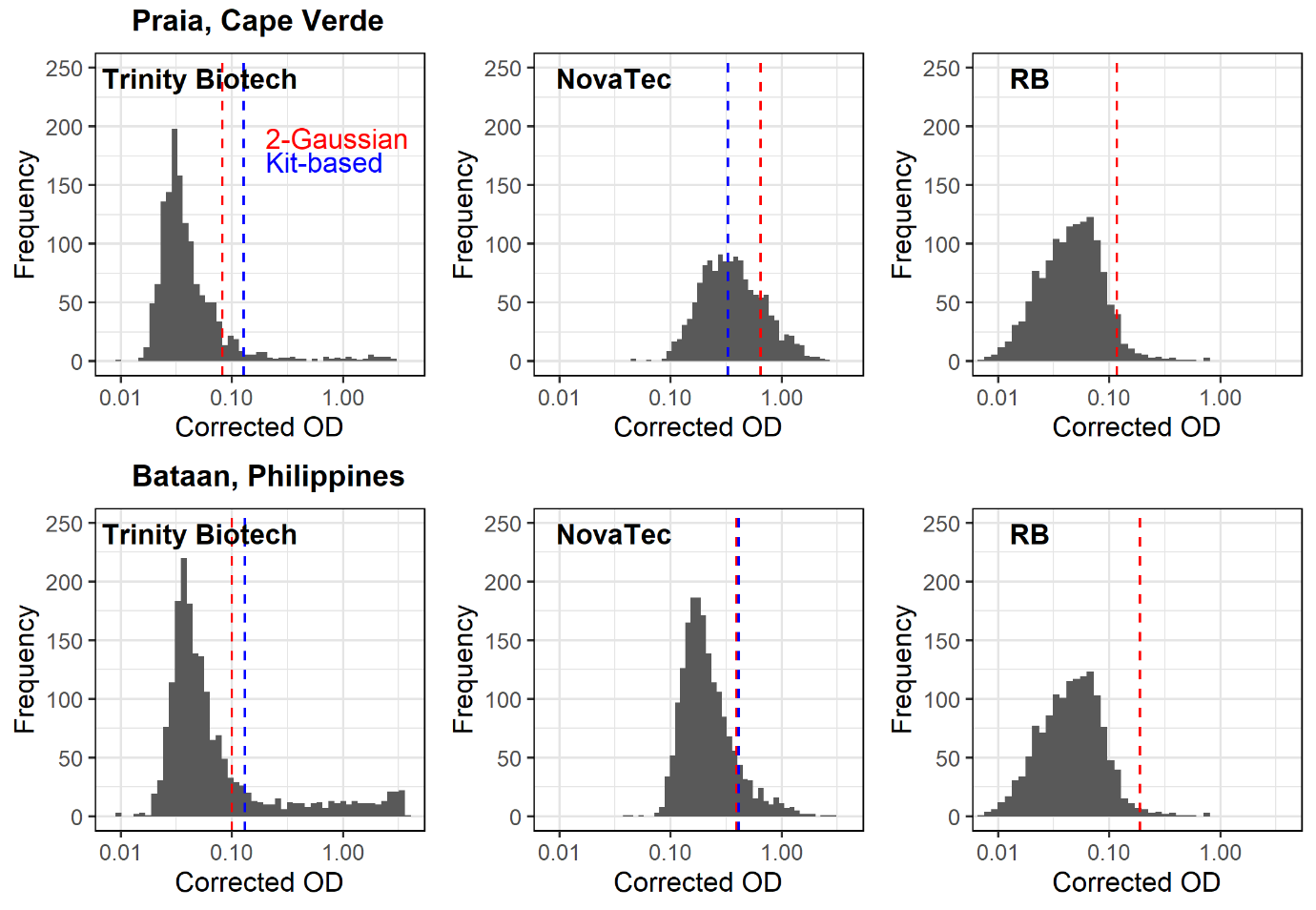
**

**Supplementary Figure 1:** **Histogram of antibody responses in Praia, Cape Verde (top) and Bataan, the Philippines (bottom) as recorded by two commercial and the research-based enzyme-linked immunosorbent assays for antimalarial antibody detection as well as thresholds for seropositivity according to different methods.** Dashed vertical lines represent thresholds for seropositivity. Thresholds according to the two-Gaussian mixture model were set at the mean of the lower distribution plus three standard deviations (red). Thresholds according to the kit instruction manual were plate-specific, the median threshold value is shown on the plots (blue). The median and range of threshold values over all plates for Trinity Biotech was 0.13 (0.12-0.14) in Praia and 0.13 (0.12-0.14) in Bataan, and for NovaTec 0.33 (0.29-0.42) in Praia and 0.41 (0.31-0.51) in Bataan.

**
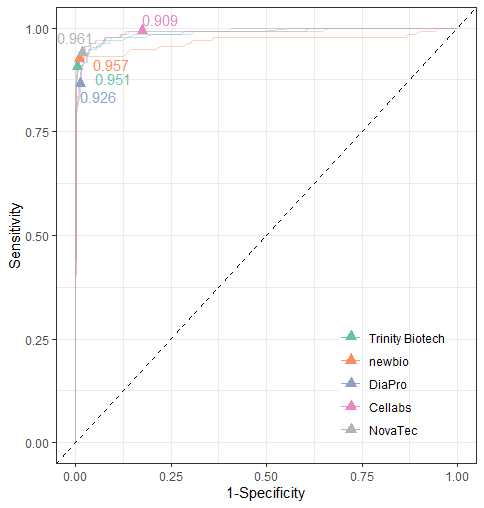
**

**Supplementary Figure 2: Receiver operating characteristic curves for commercial enzyme-linked immunosorbent assays for antimalarial antibody detection.** Sera from malaria-naïve only (n=223), and malaria-naïve and *Toxoplasma*-exposed (n=191) individuals was used to represent true negatives, while hyperimmune sera from individuals living in Farafenni, the Gambia was used to represent true positives (n=134). For the latter, sera were collected in the early 1990s and individuals were only included if they were 10 years or older by which exposure to malaria almost certainly would have occurred. The dashed diagonal line represents the reference line. Solid lines represent results for continuous antibody response data (i.e., optical density, OD) while triangles represent results for seropositivity. Area under the curve (AUC) values for seropositivity are shown on the plot.

**
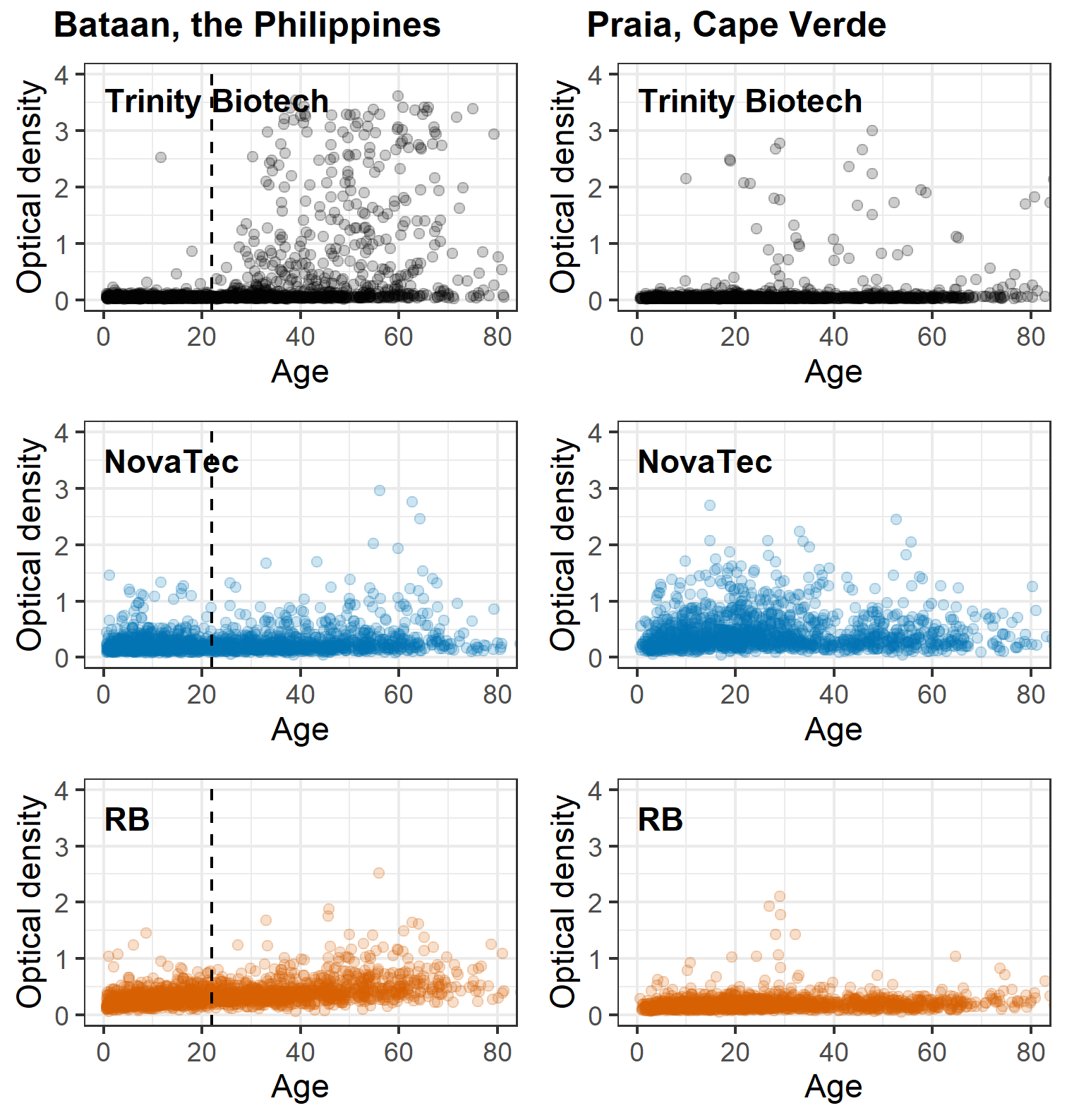
**

**Supplementary Figure 3: Optical density measurements by age as recorded by two commercial and the research-based (RB) enzyme-linked immunosorbent assays for antimalarial antibody detection.** Dots are jittered and represent individual measurements for 1,824 participants in Bataan, the Philippines and 1,396 participants in Praia, Cape Verde. The dashed vertical line in plots for Bataan represents the timing of the marked decrease in passively detected malaria cases in 1995 (i.e., 22 years prior to data collection; see Figure 2a).

**
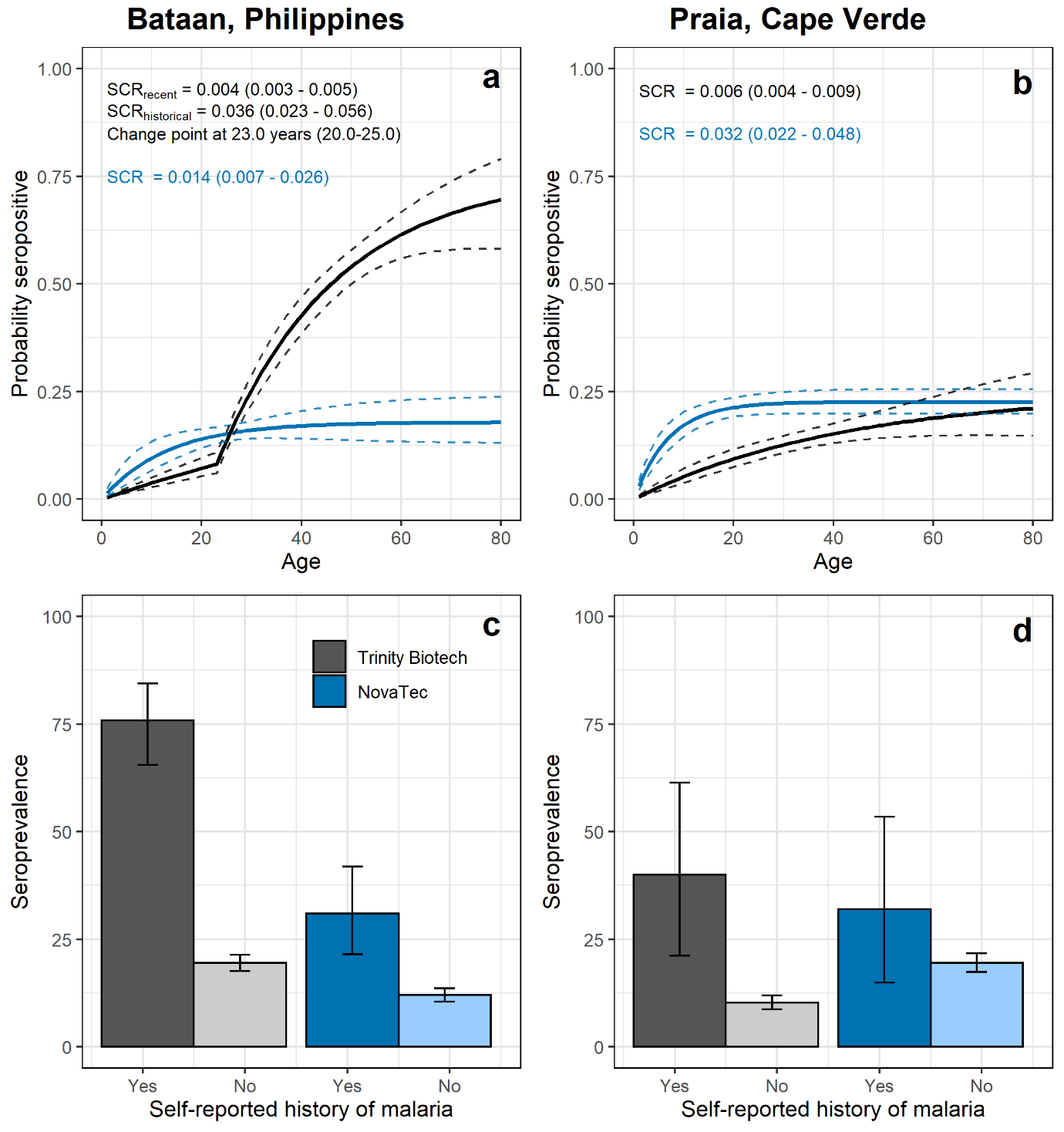
**

**Supplementary Figure 4: Seroconversion curves (a & b) and seroprevalence by self-reported history of malaria (c & d) using antibody responses recorded by commercial enzyme-linked immunosorbent assays and a two-Gaussian mixture model for seropositivity thresholds.** In (**a** & **b**) seroconversion curves of seroprevalence by age are shown; solid lines represent the fit of the reversible catalytic model (2), while dashed lines represent 95% CIs. In (**c** & **d**) seroprevalence estimates and 95% CIs are shown by categories of self-reported history of malaria. Seropositivity thresholds were set at the mean of the lower distribution plus three standard deviations using a two-Gaussian mixture model (see methods and Supplementary Figure 1).

**Supplementary Table 1: Selected and alternative seroconversion models for Cape Verde.** Final seroconversion models for the entire study population in Cape Verde are those with one force of infection; the seroconversion rate (SCR) and seroprevalence estimates for models with two forces of infection are shown when p<0.100 compared to a model with one force of infection. Similarly, for seroconversion models by gender, SCR estimates for models with two forces of infection with a change point at 20 years are shown if p<0.100 compared to a model with one force of infection. Fold-differences in seroprevalence estimates after/before the change are shown for both genders when a model with a change point showed p<0.100 for either gender. A change-point at the age of 20 by gender was investigated based on previously published data detailing an increased risk for adult men. CI: confidence interval; RB-ELISA: research-based enzyme-linked immunosorbent assay.

|  | **Entire study population**  n=1396 | | | | **By gender**  Men n=536; women n=860 | | | | |
| --- | --- | --- | --- | --- | --- | --- | --- | --- | --- |
|  | **One force of infection** | **Two forces of infection** | | | **Two forces of infection** | | | | |
|  | **SCR (95% CI)** | **Change point (95% CI),**  **p-value** | **SCR before/after change point** | **Sero-prevalence before change, n/N (%)** | **Change point, p-value** | | **Fold difference sero-prevalence after/before change** | | **SCR before/after change point** |
|  |  |  |  |  | **Men** | **Women** | **Men** | **Women** | **Men** |
| **Trinity Biotech** | | | | | | | | | |
| - FMM | 0.0059  (0.0039-0.0089) | 2 (2-12) p=0.009 | 0.0196/0.0030 | 1/26  (3.7%) | 20 p=0.337 | 20  p=0.208 | N/A | N/A | N/A |
| - Kit | 0.0025  (0.0020-0.0030) | 63 (55.5-74.5) p<0.001 | 0.0027/0.0965 | 68/1309 (5.2%) | 20  p=0.026 | 20  p=0.680 | 6.78 | 2.16 | 0.0025/0.1214 |
| **NovaTec** | | | | | | | | | |
| - FMM | 0.0323  (0.0218-0.0478) | 3 (2-15) p=0.025 | 0.0000/0.1211 | 0/54  (0.0%) | 20 p=0.118 | 20  p=0.438 | N/A | N/A | N/A |
| - Kit | 0.1283  (0.0959-0.1716) | 14.5  p=0.205 | N/A | N/A | 20 p=0.409 | 20  p=0.655 | N/A | N/A | N/A |
| **RB-ELISA** | | | | | | | | | |
| - FMM | 0.0030  (0.0014-0.0067) | 3 (2-22) p=0.090 | 0.0068/0.0009 | 1/54  (1.9%) | 20 p=0.060 | 20  p=0.564 | 3.76 | 1.63 | 0.0045/0.2180 |
